# Sorbs1 and -2 Interact with CrkL and Are Required for Acetylcholine Receptor Cluster Formation

**DOI:** 10.1128/MCB.00775-15

**Published:** 2016-01-04

**Authors:** Peter T. Hallock, Sherry Chin, Steven Blais, Thomas A. Neubert, David J. Glass

**Affiliations:** a Novartis Institutes for Biomedical Research, Cambridge, Massachusetts, USA; b Skirball Institute of Biomolecular Medicine, New York University School of Medicine, New York, New York, USA

## Abstract

Crk and CrkL are noncatalytic adaptor proteins necessary for the formation of neuromuscular synapses which function downstream of muscle-specific kinase (MuSK), a receptor tyrosine kinase expressed in skeletal muscle, and the MuSK binding protein Dok-7. How Crk/CrkL regulate neuromuscular endplate formation is not known. To better understand the roles of Crk/CrkL, we identified CrkL binding proteins using mass spectrometry and have identified Sorbs1 and Sorbs2 as two functionally redundant proteins that associate with the initiating MuSK/Dok-7/Crk/CrkL complex, regulate acetylcholine receptor (AChR) clustering *in vitro*, and are localized at synapses *in vivo*.

## INTRODUCTION

Synaptic differentiation at the neuromuscular synapse is controlled by a receptor called the muscle-specific kinase (MuSK) and the MuSK binding protein downstream-of-kinase-7 (Dok-7) (1–3). Mice lacking either MuSK or Dok-7 fail to form neuromuscular synapses, succumb at birth due to an inability to breathe, and are phenotypically indistinguishable (1, 3). MuSK was identified as part of the receptor complex for nerve-derived agrin, the secreted protein that initiates neuromuscular junction (NMJ) formation (4). Dok-7 binds phosphorylated MuSK and is both necessary and sufficient for full catalytic activity of MuSK (5, 6). Once activated, MuSK phosphorylates substrates necessary for critical aspects of both pre- and postsynaptic differentiation, including the clustering of acetylcholine receptors (AChRs) in apposition to the motor nerve terminal (7–9). The signaling events that occur immediately downstream of MuSK activation are poorly understood but require the C-terminal domain of Dok-7, which contains two conserved sites of tyrosine phosphorylation (1, 10, 11). The two Dok-7 tyrosine phosphorylation sites conform to consensus Src homology 2 (SH2) binding motifs and tether the Crk (CT10 regulator of kinase) family of SH2-containing proteins to the MuSK/Dok-7 complex (10, 12). Conditional genetic loss-of-function experiments in mice that reduce Crk/CrkL protein levels in muscle severely perturb synapse formation, causing neonatal lethality and a failure to breathe (12). These findings suggest that a MuSK/Dok-7/Crk signaling complex enables critical aspects of synaptic differentiation.

Hypomorphic mutations in Dok-7 give rise to a form of congenital myasthenic syndromes (CMS) called limb-girdle myasthenia in humans. Dok-7 limb-girdle patients harbor small and simplified synapses that lead to muscle weakness and fatigue (13–15). The most prevalent CMS Dok-7 allele, the 1124_1127dupTGCC mutation, produces a C-terminally truncated form of Dok-7 that abolishes the Crk/CrkL binding sites (13), and homozygous 1124_1127dupTGCC Dok-7 knock-in mutant mice form functionally defective synapses which result in early postnatal lethality (11). Because truncated Dok-7 encoded by the 1124_1127dupTGCC allele retains the ability to stimulate MuSK kinase activity, these data demonstrate a critical role for the C-terminal domain of Dok-7 both in mice and in humans with disease (13). How Crk and CrkL mediate synaptic differentiation, once recruited to phosphorylated Dok-7, is not known.

The Crk family of signaling adaptors is composed of two proteins, Crk and CrkL, which lack catalytic activity and function primarily by facilitating protein-protein interactions via a single N-terminal SH2 domain and two C-terminal Src homology 3 (SH3) domains (16–18). Crk/CrkL proteins have been implicated in a broad range of cellular functions, including but not limited to clear roles in integrating signals from growth factors and extracellular matrix molecules and in regulation of cell adhesion and migration, cellular proliferation, apoptosis, and even gene expression (16, 19). Despite lacking catalytic activity, Crk/CrkL adaptors are regulated by autoinhibitory tyrosine phosphorylation sites on Crk/CrkL which facilitate an intramolecular interaction that is sufficient to block adaptor function (20–22). Interestingly, Crk/CrkL proteins are tyrosine phosphorylated in response to MuSK activation, in a manner dependent on the Abelson family of nonreceptor tyrosine kinases, raising the possibility that negative regulatory mechanisms are utilized by the MuSK signaling pathway to control Crk/CrkL function (23). Identifying additional Crk/CrkL SH3 binding proteins and elucidating a functional role for these interacting proteins, with respect to synaptic differentiation, would provide a better understanding of MuSK signaling and potential insight into synaptic dysfunction in human disease.

Here we show that knockdown of Crk and Crk proteins severely inhibits the formation and maturation of AChR aggregates *in vitro*. Further, we reconstituted tagged forms of CrkL and identified CrkL binding proteins in muscle cells using mass spectrometry (MS). Finally, we screened CrkL binding proteins using RNA interference (RNAi) and identified Sorbs1 and Sorbs2 as two proteins that are enriched at AChR clusters and are likewise required for the formation of AChR aggregation *in vitro*.

## MATERIALS AND METHODS

### Cell culture.

Murine C2C12 myoblasts were obtained from the American Type Culture Collection (ATCC) and cultured in Dulbecco modified Eagle medium (DMEM) (Gibco) supplemented with 10% fetal bovine serum (FBS) (HyClone), 4.5 g/liter d-glucose, 4 mM l-glutamine, and penicillin-streptomycin. To induce myoblast differentiation, C2C12 myoblasts were grown to confluence (day 0) and culture medium was changed to DMEM supplemented with 2% horse serum (HyClone), 4.5 g/liter d-glucose, 4 mM l-glutamine, and penicillin-streptomycin. Differentiation serum was prepared fresh for all experiments. Myoblast differentiation was largely complete by day 3, and myoblasts were transfected with small interfering RNA (siRNA) and analyzed on day 5.

### Immunofluorescence.

AChR aggregates were visualized by incubating myotubes with a solution containing 1.0 μg/ml alpha-bungarotoxin–Alexa Fluor 594 in DMEM for 15 min at 37°C with 7.5% CO_2_. Cells were rinsed twice with warmed phosphate-buffered saline (PBS) and fixed immediately in a 4% paraformaldehyde (PFA)–PBS solution. After 30 min at room temperature in fixative, cells were rinsed with PBS and permeabilized using 0.5% Triton X-100 in PBS for 10 min. Permeabilization buffer was flushed out by three PBS rinses, and myotubes were blocked with a 4% bovine serum albumin (BSA)–PBS solution for 1 h at room temperature. Next, samples were incubated with a primary antibody at 4°C overnight in blocking buffer. Primary antibody solutions were washed out using a minimum of five PBS rinses and incubated for a minimum of 2 h at room temperature using a secondary antibody conjugated to Alexa Fluor 488 specific to the host primary antibody. In this study, we used antibodies to Sorbs1 (Abcam ab4551) and Sorbs2 (S5C Sigma) to label endogenous Sorbs1 or -2 protein in combination with donkey anti-goat antibody–Alexa Fluor 488 (Invitrogen A11055) or goat anti-mouse antibody–Alexa Fluor 488 (Invitrogen A11001). Additionally, anti-green fluorescent protein (GFP)–Alexa Fluor 488 (Invitrogen A21311) was used to potentiate GFP fluorescence after PFA fixation.

### Generation of Sorbs1 and Sorbs2 mutant C2C12 cell lines.

Early-passage C2 myoblasts were transiently transfected with plasmid DNA containing a U6 promoter driving guide-RNA transcripts and a cytomegalovirus (CMV) promoter driving expression of wild-type type II Cas9 fused in frame with a T2A-GFP cassette. We targeted internal exons for both Sorbs1/2 that were predicted to disrupt all coding transcripts using the seed sequences CAGAACTCACTTCGCCCGGC (Sorbs1) and CTCTTTAACAGACTGTCGAC (Sorbs2). GFP-positive myoblasts were isolated by fluorescence-activated cell sorting (FACS) and plated out at low density for subcloning, and mutations at each locus were confirmed by a T7 endonuclease digestion assay (NEB) using the primer pairs 5′-TCAGCTCAGCCATGGTAACAC/5′-CTGGTAGACGCCCTCATCAG for Sorbs1 and 5′-CATAATCATAGCAAGATGTCTGCC/5′-GAACAATACCCGAGTGCAGATG for Sorbs2. Individual clones were screened for their ability to differentiate into myotubes, and amplicons from the targeted locus of differentiating cells were screened for the presence of indel mutations using a T7 endonuclease assay. To identify biallelic loss-of-function mutations in individual clones, amplicons to the targeted locus were inserted into sequencing vectors using TOPO-TA (Invitrogen) cloning, and a minimum of 10 individual vectors were subjected to Sanger sequencing. Biallelic loss-of-function mutations in each gene were confirmed by Western blotting with antibodies to Sorbs1 (Abcam ab4551) or Sorbs2 (Sigma S5C).

### RNAi knockdown assay.

Myoblasts were differentiated on a Matrigel- (or laminin-111)-coated 96-well plate. Myotubes were treated with RNAiMAX (Invitrogen) loaded with a nontargetable siRNA (AM4626; Ambion) or siRNAs against individual transcripts (Qiagen) at a final concentration of 20 nM for 4 h. Transfection medium was replaced with fresh differentiation medium, and cells were cultured for 48 h. Myotubes were stained with alpha-bungarotoxin-conjugated Alexa Fluor 594 (Life Technologies), fixed in 4% paraformaldehyde in PBS, and imaged using an Arrayscan Vti high-content imager (Cellomics). Montages containing 16 fields at a magnification of ×10 were analyzed with ImageJ software (NIH), and AChR cluster number and myotube area were quantified using intensity threshold gating.

### *In vitro*-transcribed mRNA and transfection.

We synthesized a cassette containing the mouse CrkL cDNA containing an N-terminal tandem affinity purification (TAP) tag and a C-terminal internal ribosome entry site (IRES)-GFP reporter element. The cassette was cloned into a shuttle vector containing a 5′ T7 polymerase binding site and a 3′ simian virus 40 (SV40) poly(A) termination signal. mRNA was synthesized by linearizing the template vector by restriction endonuclease digestion 3′ to the SV40 poly(A) sequence. TAP-tagged mRNAs were transcribed using mMessage-mMachine (Life Technologies), mRNA was purified using a MEGAclear column (Life Technologies), and discrete band size was confirmed by agarose gel electrophoresis. Myotubes were transfected with mRNAs using Lipofectamine 2000 (Life Technologies), and protein expression was confirmed by Western blotting using antibodies to CrkL (C-20; Santa Cruz Biotechnology) or CrkI/II (monoclonal antibody [MAb] 22; BD Biosciences).

### Western blot analysis.

C2C12 myotubes were lysed by mechanical scraping of petri dishes in nonionic detergent buffer containing 1% Nonidet P-40, 50 mM Tris-HCl (pH 7.4), 150 mM NaCl, 5 mM EDTA, 2 mM sodium orthovanadate, and complete protease inhibitors (Roche). Homogenates were centrifuged at 13,000 rpm for 15 min at 4°C, and total protein from supernatants was quantified using a bicinchoninic acid kit (Pierce). MuSK was immunoprecipitated using rabbit antiserum against the C terminus of MuSK, a kind gift from Steven Burden (NYU), or immunoblotted with antibodies to the extracellular domain of MuSK (AF3904; R&D Systems). Dok-7 was immunoprecipitated with rabbit antiserum directed against the C terminus (12) or immunoblotted with Dok-7 antiserum (AF6398; R&D Systems). Monoclonal antibodies directed against phosphotyrosine (4G10) were from Millipore. Immunoprecipitates or lysates were reduced using SDS-PAGE sample buffer (Boston Bioproducts) and denatured for 5 min at 65°C. Samples were loaded into a 4 to 12% Bis-Tris NuPage gel, electrophoresed, and transferred to a polyvinylidene difluoride (PVDF) membrane. Membranes were blocked in 4% bovine serum albumin (BSA) in Tris-buffered saline (TBS) containing 0.05% Tween 20 (TBST). Primary antibodies were incubated overnight at 4°C in TBST containing 4% BSA. The following day, membranes were rinsed in excess TBST and incubated with an appropriate horseradish peroxidase (HRP)-conjugated or infrared dye-conjugated secondary antibody. Immunoreactivity was visualized directly using a LiCor Odyssey infrared imager or by chemiluminescence using an ECL Plus Western blotting substrate (Pierce). An additional antibody used in this study was anti-histone H3 (Cell Signaling) as a loading control.

### Histology.

All procedures involving animals were approved by the Institutional Animal Care and Use Committee of the Novartis Institutes for Biomedical Research, Cambridge, MA. Adult C57BL/6J mice were housed individually with environmental enrichment in a temperature (22°C)- and humidity (43%)-controlled room. Mice were maintained on a 12-h light-dark cycle and provided food (5053, PicoLab Rodent Diet 20; LabDiets) and water *ad libitum*. Gastrocnemius, soleus, and plantaris muscles were harvested and snap-frozen in liquid nitrogen cooled with isopentane. Muscle samples were sectioned at a 10-μm thickness, dehydrated, and stored at −80 until staining.

### Mass spectrometry.

Control and TAP-CrkL samples were separated by SDS-PAGE and visualized by silver staining (LC6070; Invitrogen). Each lane was cut into 17 equal individual slices without regard to the staining pattern. The samples were destained, reduced with 20 mM dithiothreitol (DTT), and alkylated with 50 mM iodoacetamide. The samples were then digested overnight with 0.1 μg trypsin per gel slice. Tryptic peptides were extracted, dried under a vacuum, and then resuspended in 12 μl 0.1% formic acid. Eight microliters of each sample was loaded onto a 75-μm by 12-cm column self-packed with 3 μm ReproSil-Pur C_18_-AQ beads (Dr. Maisch GmbH), eluted with a gradient of 2 to 40% acetonitrile in 0.1% formic acid over 50 min at 300 nl/min, and analyzed using a Q-Exactive mass spectrometer (Thermo Fisher Scientific). Proteins were identified using the Andromeda search engine (MaxQuant version 1.2.2.5) with cysteine carbamidomethylation specified as a fixed modification and methionine oxidation as a variable modification to search the Swiss-Prot database. Relative quantities of the proteins were determined using the iBAQ feature of MaxQuant. The entire data set is included as Data Set S1 in the supplemental material, and the raw data are hosted at ftp://massive.ucsd.edu/MSV000079311/.

### Analysis of putative CrkL binding proteins.

To determine whether a CrkL-associated protein could bind CrkL we analyzed the primary amino acid sequences of CrkL-associated proteins using Scansite 2.0 (http://scansite.mit.edu/) at medium stringency and scored each protein in Table S1 in the supplemental material on whether it was predicted to interact with the Crk SH2 domain, the Crk SH3 domain, or both domains. A fraction of the CrkL-associated proteins identified in our tandem mass spectrometry (MS/MS) data set have been confirmed by more rigorous experimentation in the literature and were classified as “known interactors.”

## RESULTS

### Crk and CrkL are required for AChR aggregation and maturation *in vitro*.

**FIG 1:**
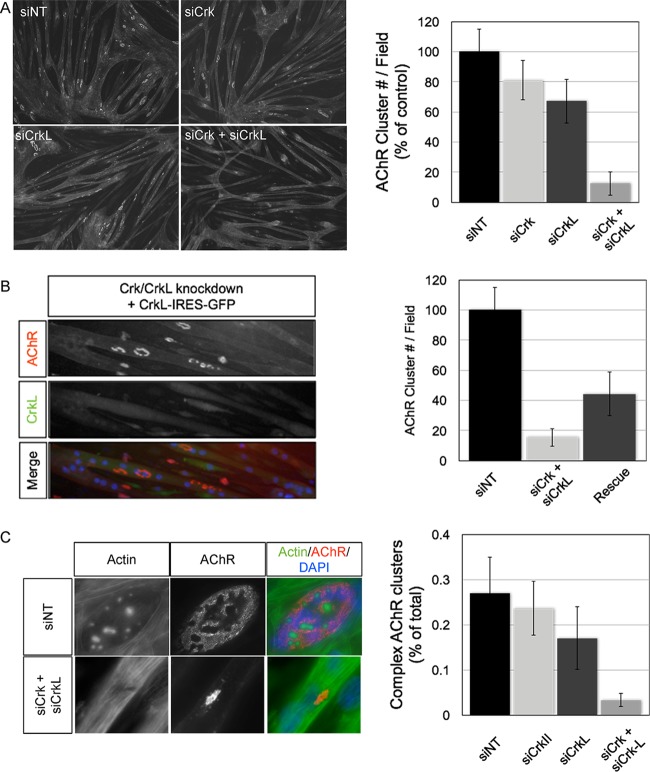
Crk/CrkL RNAi-treated myotubes display AChR clusters that are fewer and lack complexity. (A) Myotubes plated on laminin-111 were transfected with siRNAs directed against a nontargetable control transcript (NT), Crk, CrkL, or both Crk and CrkL. At 48 h posttransfection, myotubes were stained with fluorophore-conjugated bungarotoxin to label AChR aggregates and quantified by intensity threshold gating. In Crk RNAi-treated myotubes, AChR clusters were modestly reduced (∼19%) compared to those in NT-treated myotubes, Similarly, in myotubes treated with CrkL RNAi, AChR aggregates were reduced ∼33% compared to those in NT-treated myotubes. In contrast, myotubes treated with both Crk and CrkL RNAi displayed ∼88% fewer AChR aggregates than NT-treated myotubes (*n* = 3; means ± standard errors of the means [SEM] are shown; *P* < 0.05). (B) Myotubes were treated with siRNAs against an NT control or Crk/CrkL as described for panel A and then transfected 4 h later with *in vitro*-transcribed CrkL mRNA that was resistant to RNAi-mediated degradation. Myotubes transfected with both CrkL mRNA and Crk/CrkL RNAi partially rescued AChR aggregation (∼44% of NT-treated myotubes versus ∼15% for Crk/CrkL siRNA-treated myotubes) (*n* = 3; means ± SEM are shown; *P* < 0.05). (C) AChR aggregates that form in Crk/CrkL RNAi-treated myotubes are small and simplified. We scored AChR aggregates for the presence or absence of cortical actin puncta, markers of maturation, and found ∼27% of AChR aggregates from NT-treated treated myotubes harbored actin puncta and that the number was reduced to nearly zero in Crk/CrkL RNAi-treated myotubes (*n* = 3; means ± SEM are shown).

Conditional hypomorphic Crk/CrkL mutant muscles exhibit nerve overgrowth, and individual myofibers display a range of AChR aggregation defects, including no AChR clusters or small/simplified motor endplates. To confirm these findings and further investigate the roles of Crk/CrkL, we sought to establish an *in vitro* culture system where Crk/CrkL expression is reduced. We isolated primary myoblasts from double homozygous floxed Crk/CrkL muscles at embryonic day 18.5 (E18.5) and expressed Cre recombinase in these cells. Cre-expressing clones did not survive under these conditions and died shortly after Cre expression, indicating an essential role for Crk/CrkL in myoblast viability *in vitro* (data not shown). Because a knockout cell line of both genes was not feasible, we assayed whether introduction of siRNAs against Crk, CrkL, or both could significantly reduce their respective protein levels in myotubes. Indeed, muscle cells transfected with siRNAs reduced Crk and CrkL protein levels by roughly one-half at 48 h posttransfection (data not shown). We then challenged Crk/CrkL RNAi-treated myotubes to cluster AChRs on a laminin-111 substrate and quantified the number of AChR clusters in a visual field. We observed modest differences in total AChR cluster number in myotubes treated with siRNAs against Crk alone or CrkL alone compared to a nontargeting control siRNA (Fig. 1A). In contrast, myotubes treated with both Crk and CrkL siRNAs reduced AChR clustering by ∼90% (*n* = 3, *P* = 0.007 [Student's *t* test]) (Fig. 1A). To exclude the possibility of off-target effects from Crk/CrkL RNAi treatment, we sought to rescue the AChR aggregation phenotype by reconstituting RNAi-resistant CrkL mRNA into myotubes. Myotubes expressing CrkL, and therefore GFP, displayed a significant increase in AChR aggregates, which facilitated a partial rescue of the aggregation phenotype (Fig. 1B). In cells treated with nontargeting siRNAs, AChR aggregates were large, and a significant percentage of aggregates contained perforations and/or branches within the aggregate that produced complex morphologies (Fig. 1C). We quantified this effect by scoring clusters for the presence or absence of cortical actin puncta, a podosome marker that underlies maturation within AChR aggregates (24, 25). In control myotubes, roughly 25% of AChR aggregates contained at least a single cortical actin punctum (Fig. 1C). In contrast, AChR clusters on Crk/CrkL knockdown myotubes were small and simplified in appearance and only in exceedingly rare occurrences (∼3%) contained cortical actin puncta (Fig. 1C). Together, these data demonstrate that Crk/CrkL adaptors are critical regulators of AChR cluster aggregation and maturation of AChR aggregates *in vitro*.

### Identification of CrkL binding proteins in myotube cultures.

Crk and CrkL assemble signaling complexes by binding tyrosine-phosphorylated residues via the Src homology 2 domain and recruiting additional proteins via two Src homology 3 domains. Because Crk adaptors bind tyrosine-phosphorylated Dok-7, we were interested in identifying SH3-dependent Crk binding proteins in myotubes to gain a better understanding of how Crk/CrkL functions in the MuSK signaling pathway.

**FIG 2:**
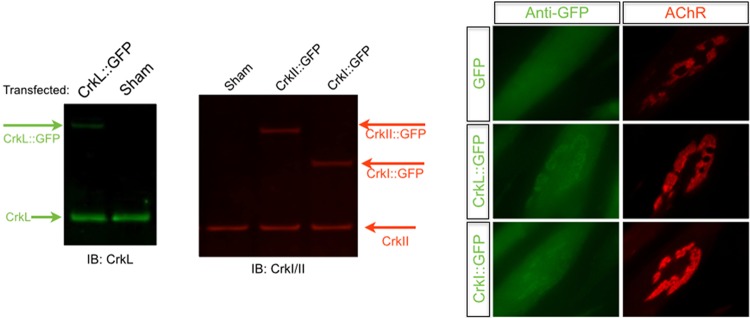
Exogenous Crk and CrkL are both enriched within AChR aggregates. *In vitro*-transcribed mRNAs encoding CrkL or both isoforms of the Crk gene, CrkI and CrkII, were fused to GFP and transfected into differentiated C2C12 myotubes. We analyzed soluble cellular lysates at 12 h posttransfection and confirmed the predicted sizes of our GFP fusion proteins by LiCor Odyssey infrared imaging using antibodies specific for CrkL or Crk. In parallel experiments, myotubes plated on laminin-111 were stained with fluorophore-conjugated bungarotoxin to label AChRs as well as Alexa Fluor 488-conjugated antibodies directed against GFP. Significant enrichment of CrkL-GFP, CrkI-GFP, and CrkII-GFP was observed within the area occupied by clustered AChRs and to a lesser degree the gaps within AChR clusters.

**FIG 3:**
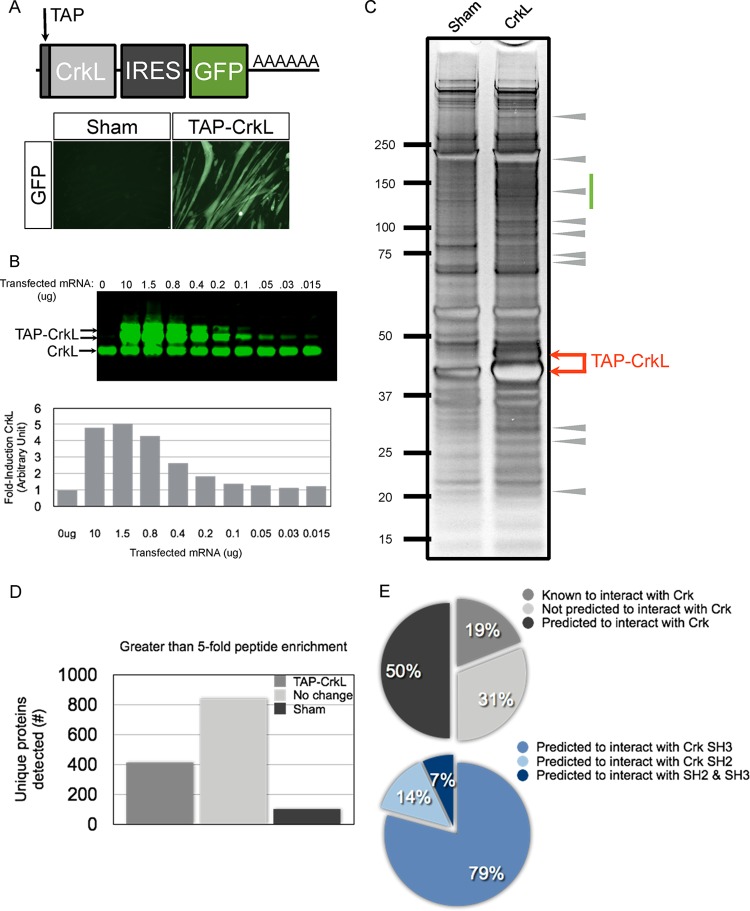
Identification of CrkL binding proteins from myotube lysates. (A) Cartoon representation of the TAP-tagged CrkL transcript that was tested. The transcript contains an N-terminal TAP tag, the CrkL coding sequence, an IRES-GFP, and an SV40 poly(A) sequence. (B) Myotubes transfected with serial dilutions of TAP-CrkL RNA and cell lysates were analyzed at 18 h posttransfection. CrkL RNA scales linearly over a 5-fold range and permits reconstitution of exogenous CrkL at levels similar to physiological CrkL protein levels. (C) TAP-CrkL proteins were purified from myotube lysates and subjected to SDS-PAGE and silver staining. The entire lane from either the sham or TAP-CrkL condition was cut into 18 individual bands and subjected to MS/MS analysis. Arrows indicate the size of the TAP-CrkL doublet band. The green bar indicates an area of the gel that was enriched with CrkL-specific interactors. Arrowheads indicate clear CrkL-specific proteins. (D) MS/MS summary of proteins identified under the two conditions. There were roughly 1,500 proteins in total, of which ∼400 proteins showed a >5-fold peptide enrichment specific to the TAP-CrkL condition. (E) Pie chart summary of CrkL-interacting proteins, summarizing proteins known, predicted, and not predicted to interact with CrkL. Of the predicted interacting proteins, many are predicted to interact with the SH3 domains of CrkL (79%).

We first sought to determine whether tagged forms of Crk/CrkL would localize within AChR aggregates, the site where phosphorylated Dok-7 is enriched. We synthesized mRNAs encoding GFP alone and GFP fused to CrkI, CrkII, and CrkL. Myotubes were transfected with mRNA, and GFP was visualized at 12 h posttransfection. As expected, we observed colocalization of Crk-GFP and CrkL-GFP fusions at sites where AChRs aggregate, indicating that tagged versions of either adaptor localize properly at sites of AChR aggregation (Fig. 2). To identify CrkL binding proteins in myotubes, we engineered a tagged form of CrkL that contains a tandem affinity purification (TAP) tag at the N terminus and an IRES-GFP reporter. Myotubes transfected with TAP-CrkL fluoresce rapidly after transfection, and fluorescence persisted over a 24-hour period (Fig. 3A). To limit the possibility of nonphysiological interactions with the bait protein, we reconstituted TAP-CrkL at levels similar to those of endogenous CrkL protein (Fig. 3B).

We purified TAP-CrkL from transfected myotube lysates, fractionated by gel electrophoresis, and visualized the CrkL precipitate by silver staining (Fig. 3C). As expected, we observed an abundant protein doublet at the predicted molecular weight of TAP-CrkL as well as many CrkL-specific binding proteins throughout the gel lane (Fig. 3C). Gels for both control and TAP-CrkL conditions were cut into 17 gel pieces, digested with trypsin, and subjected to mass spectrometric analysis, and peptide abundance was determined using intensity-based absolute quantification (IBAQ). We identified roughly 1,300 proteins total, of which approximately 400 proteins exhibited >5-fold peptide enrichment under the TAP-CrkL condition compared to the control (Fig. 3D; see Data Set S1 in the supplemental material). Among these proteins are those with roles in translation (ribosomal proteins), sequestration (heat shock proteins), and degradation (proteasome components). Importantly, we were able to purify endogenous Dok-7, a positive control, indicating that TAP-CrkL associates with the upstream MuSK/Dok-7 complex. We next curated the list of CrkL binding proteins to the top 61 hits, representing 15% of the total, after removing candidates with clear roles in protein homeostasis (see Table S1 in the supplemental material). Analysis of the top hits identified 11 previously characterized Crk/CrkL binding proteins (e.g., Dok-7, Abl1, Rapgef1, and DOCK1) and 30 proteins predicted to interact with Crk/CrkL (e.g., Arhgef5, Sgk269, Tks5, and utrophin), as well as 20 proteins not predicted to interact with Crk/CrkL (e.g., coronin-6, Elmo2, and LL5beta) (Fig. 3E; see Table S1 in the supplemental material). Of the top 61 hits, most proteins (∼86%) were predicted or known to interact with the SH3 domains of Crk/CrkL (Fig. 3E). We verified a subset of these putative CrkL-interacting proteins by cotransfection in 293 cells (data not shown). Together, these data demonstrate that reconstituted TAP-CrkL binds Dok-7 and copurifies with many proteins predicted to bind the Crk/CrkL Src homology 3 domains, which are likely to contain candidates that play important roles in mediating MuSK signaling.

### Sorbs1 is enriched at AChR aggregates, and loss of Sorbs1 inhibits AChR cluster formation in muscle cells.

**FIG 4:**
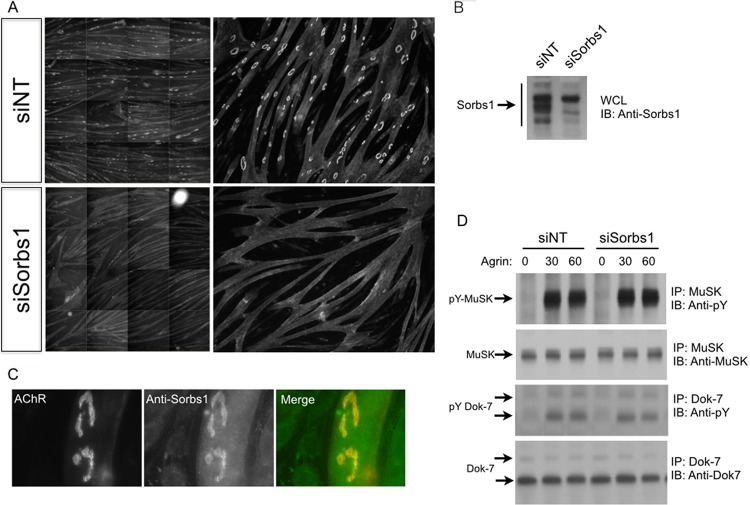
Sorbs1 is enriched at AChR aggregates, and Sorbs1 RNAi blocks AChR clustering *in vitro*. (A) Treatment of myotubes with siRNA directed against Sorbs1 blocks AChR clustering in C2C12 cells. Montages containing sixteen fields at a magnification of 10× were analyzed with ImageJ software (NIH). (B) Sorbs1 siRNA significantly reduces Sorbs1 protein expression in myotubes. (C) Sorbs1 protein is highly enriched at sites where AChRs aggregate. (D) Agrin stimulates tyrosine phosphorylation of MuSK and Dok-7 at similar levels in myotubes treated with Sorbs1 siRNA.

**FIG 5:**
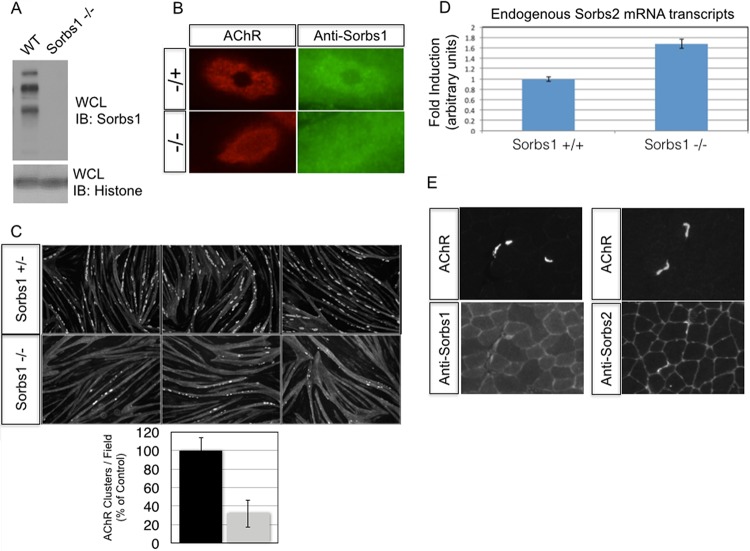
Reduced AChR aggregation in a Sorbs1 knockout cell line. (A) Sorbs1 mutant C2C12 myotubes lack protein expression of all splice variants of Sorbs1 in myotubes. (B) Protein enrichment of Sorbs1 at AChR aggregates is specific to Sorbs1, as antibodies fail to label AChR aggregates in Sorbs1 mutant cells. (C) AChR aggregates are reduced 3-fold in Sorbs1 mutant cells. (D) Relative expression of Sorbs2 is upregulated 1.7-fold in Sorbs1 myotubes compared to wild-type control (*n* = 3, means ± SEM are shown). (E) A single muscle section was stained with bungarotoxin-Alexa Fluor 594 and an antibody to Sorbs1 or Sorbs2. Sorbs1/2 protein enrichment is detected at adult murine synapses *in vivo*.

To identify Crk/CrkL binding proteins that are required for AChR aggregation, we assayed candidate genes using RNAi in combination with high-content imaging. We plated myoblasts on laminin-coated 96-well plates, transfected myotubes with siRNA-loaded liposomes, and assayed the ability of these cells to form AChR aggregates on laminin at 48 h after siRNA treatment. As expected, treatment of myotubes with siRNAs directed against MuSK, Lrp4, or the AChR β subunit reduced AChR aggregate formation (data not shown). Interestingly, knockdown of Sorbs1 in myotubes severely inhibited the formation of AChR aggregates compared to that in control nontargeting siRNA-treated cells (Fig. 4A). Sorbs1/2 were both identified in our MS/MS pulldown data set (see Table S1 in the supplemental material for evidence for Sorbs2 and Data Set S1 in the supplemental material for evidence for Sorbs1). Next, we assayed whether Sorbs1 siRNAs reduce Sorbs1 transcripts (data not shown) and probed whole-cell lysates to confirm that Sorbs1 protein levels were likewise significantly reduced (Fig. 4B). Having identified an antibody that recognizes mouse Sorbs1, we probed the subcellular localization of Sorbs1 and observed strong antibody labeling enriched within the AChR-rich membrane (Fig. 4C). Because of Sorbs1 protein localization and the phenotypic severity of loss of Sorbs1 on AChR aggregation, we tested whether knockdown of Sorbs1 might affect expression levels of MuSK and/or MuSK activation. We stimulated Sorbs1 knockdown myotubes with neural agrin, precipitated MuSK, and Western blotted for total MuSK and tyrosine-phosphorylated MuSK. Total MuSK expression was comparable across all conditions, and neural agrin elicited a striking increase in tyrosine-phosphorylated MuSK (Fig. 5D). Moreover, the total expression levels of Dok-7 and the extent of Dok-7 tyrosine phosphorylation were likewise similar between the Sorbs1 knockdown and control myotubes (Fig. 4D). These data indicate that Sorbs1 acts downstream of MuSK/Dok-7 to regulate the clustering of AChRs *in vitro*.

**FIG 6:**
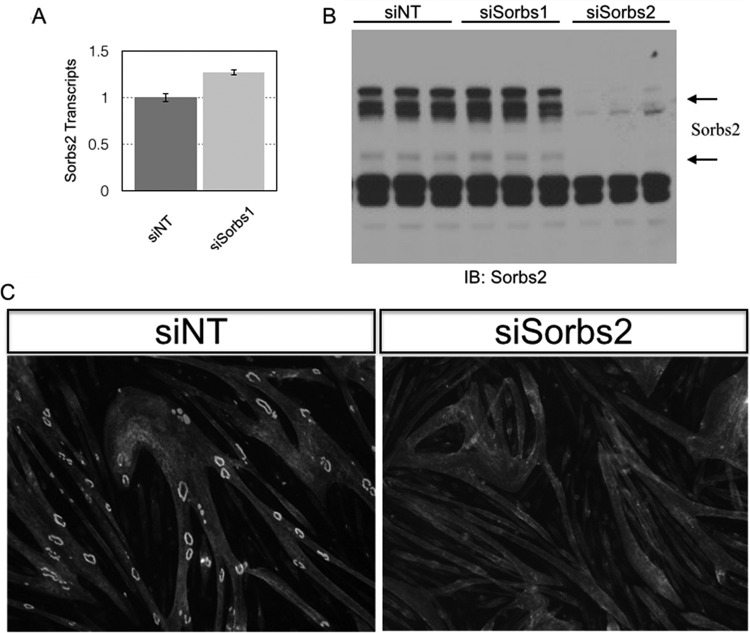
Sorbs2 knockdown inhibits AChR clustering in muscle cells. (A) Myotubes were transfected with Sorbs1 or a nontargeting siRNA, and total RNA was isolated 48 h later. Total Sorbs2 transcript levels were assessed by quantitative PCR and found to be modestly elevated (∼25%) in myotubes treated with Sorbs1 siRNAs. (B) Myotubes were transfected with siRNAs directed against Sorbs1, Sorbs2, or a nontargeting control (siScr). Cell lysates were probed with antibodies directed against Sorbs2. Sorbs2 protein is significantly reduced by Sorbs2 RNAi treatment and modestly increased with Sorbs1 RNAi treatment. (C) Knockdown of Sorbs2 protein levels inhibits AChR clustering independent of Sorbs1.

To confirm our Sorbs1 RNAi observations and validate our Sorbs1 antibody, we generated a Sorbs1 mutant C2C12 cell line using CRISPR/Cas9. The mouse Sorbs1 locus produces alternatively spliced Sorbs1 transcripts, and as a consequence we targeted an internal region of Sorbs1 that is common to all coding transcripts (see Materials and Methods). We transfected Cas9 along with a Sorbs1 guide RNA into C2 cells and subcloned from a pool of cells containing mutations in the targeted locus of Sorbs1. Individual clones were screened for myotube differentiation before Sanger sequencing confirmed biallelic loss-of-function mutations at the Sorbs1 locus. Next, we confirmed the specificity of our Sorbs1 antibody using Western blotting of control and mutant Sorbs1 C2C12 cells. In control cells, Sorbs1 antibodies labeled multiple high-molecular-weight bands, but they failed to label these same bands in our Sorbs1 mutant cell line (Fig. 5A). Next, we confirmed the specificity of the Sorbs1 subcellular localization data using our Sorbs1 mutant cell line. We observed weak staining at AChR aggregates in a Sorbs1 heterozygous cell line but failed to detect appreciable staining at AChR aggregates in the Sorbs1 mutant muscle cell line (Fig. 5B). Finally, we challenged the Sorbs1 mutant myotubes to form AChR clusters to validate our RNAi knockdown experiments. Surprisingly, we found that AChR clustering was reduced compared to that in our control cell line but that overall these cells were better capable of clustering AChRs than Sorbs1 RNAi-treated myotubes (Fig. 5C). Compensatory mechanisms might explain the weaker phenotype in Sorbs1 mutant myotubes compared to Sorbs1 RNAi-treated myotubes. In support of this model, we quantified the levels of a second Sorbs family member, Sorbs2, and found that total Sorbs2 transcripts were significantly increased in Sorbs1 mutant myotubes compare to control myotubes (Fig. 5D). Because we observed an increase of Sorbs2 mRNA transcripts in the Sorbs1 mutant background, we treated wild-type C2 myotubes with Sorbs2 RNAi and challenged these myotubes to form AChR aggregates. Indeed, knockdown of myotubes with Sorbs2 siRNAs severely perturbed AChR cluster formation (Fig. 6A to C). Because both Sorbs1 and Sorbs2 RNAi inhibited AChR aggregation and Sorbs2 was upregulated in the Sorbs1 mutant cell line, we sought to generate a double Sorbs1/2 mutant cell line. We transfected Sorbs1 mutant cells and control cells with guide RNAs directed against the sorbin homology (SoHo) domain of Sorbs2. We recovered Sorbs2 mutant cells from the wild-type line but failed to recover double mutant cells from the Sorbs1 mutant experimental line (data not shown). Together these data suggest that Sorbs1/2 are necessary for myoblast viability *in vitro*.

We sought to verify whether Sorbs1 and/or Sorbs2 is enriched at synapses and stained cross sections of adult mouse gastrocnemius muscles with antibodies to Sorbs1 and Sorbs2. We observed staining of Sorbs1 and strong staining of Sorbs2 at synapses *in vivo* (Fig. 5E). Together, these data demonstrate that Sorbs1 is highly enriched at AChR aggregates *in vitro* and that loss of Sorbs1 severely perturbs AChR clustering *in vitro*. In combination with our *in vivo* staining of synapses, these data indicate that Sorbs family members may play critical roles in synaptic differentiation.

## DISCUSSION

It was previously demonstrated that Crk/CrkL adaptor proteins control the formation of synapses, and this requirement was reproduced in the current study via siRNA-mediated knockdown of Crk and CrkL, as knockdown of both Crk and CrkL was required for significant inhibition of AChR aggregation. To progress with the understanding of that requirement, in this study a large group of CrkL binding proteins were identified from cultured muscle cell lysates. A significant fraction of these proteins have previously been implicated in MuSK signaling, giving confidence in the current study. Several proteins which were not previously appreciated to play a role in synaptic differentiation were identified in this study. For example, Abl1/2, Dok-7, and utrophin were all identified in this study and have each been previously shown to play a role in synaptic differentiation (1, 23, 26). Moreover, we identified coronin-6 as a CrkL-associated protein which has been recently shown to play functional roles in AChR aggregation and maturation (27). One pair of proteins which bound CrkL, Sorbs1 and Sorbs2, had not been previously been shown to regulate AChR clustering but were shown in this study to associate with CrkL and to be differentially required for AChR clustering in myotubes.

The three-member vinexin family of adaptor proteins is comprised of Sorbs1, Sorbs2, and vinexin/Sorbs3. These adaptors are characterized by the presence of a sorbin homology (SoHo) domain, three SH3 domains, and multiple polyproline motifs, all of which facilitate protein-protein interactions. Interestingly, Sorbs1 and Sorbs2 contain an additional homologous region that is not conserved within vinexin/Sorbs3, suggesting that Sorbs1 and Sorbs2 are more closely related. Previous studies have shown that Sorbs proteins play important roles in growth factor-induced signal transduction, cell adhesion, and cytoskeletal organization. Based on our mass spectrometric analysis, the MuSK/Dok-7/Crk complex interacts with a large body of known regulators of cytoskeletal dynamics. How might Crk/CrkL recruitment of Sorbs1/2 engage the actin cytoskeleton? Vinculin is a well-characterized Sorbs1/2 binding protein which binds directly to actin and plays key roles in actin reorganization. These data raise the possibility that a Crk/Sorbs/vinculin complex reorganizes actin at sites where MuSK is activated.

Knockdown of both Crk and CrkL is necessary to significantly inhibit AChR clustering both *in vitro* and *in vivo*. In contrast, knockdown of either Sorbs1 or Sorbs2 severely inhibits AChR aggregation *in vitro*. Our Sorbs1 RNAi data were confirmed in Cas9-mediated knockout muscle cells, since we observed an ∼3-fold reduction in AChR aggregates in myotubes devoid of Sorbs1 protein. What seems to be mitigating the complete loss of AChR aggregates in Sorbs1 mutant myotubes is a compensatory 1.7× increase in Sorbs2 mRNA transcripts. Because RNAi knockdown of either Sorbs1 or Sorbs2 is sufficient to inhibit AChR aggregation, these data combined suggest that Sorbs1/2 may not share complete redundancy. Loss-of-function Sorbs1 mutant mice are viable, indicating that Sorbs1 is not essential for synapse formation or function (28). Whether more subtle neuromuscular synaptic defects manifest in Sorbs1 mutant mice is not clear. Ultimately, careful genetic analysis of mice lacking Sorbs1, Sorbs2, and both Sorbs1/2 in muscle will be necessary to determine what function, if any, Sorbs1/2 proteins play in neuromuscular synaptic differentiation.

Of interest, Sorbs1 has been shown to be a substrate for Abl1 (29), which itself has been identified as playing a role in clustering. The current data therefore suggest a MuSK/Dok7/Crk-CrkL/Sorbs1/2 pathway, with Abl phosphorylation potentially playing a role in either recruitment of Sorbs1 or its status as a further binding site for other proteins at the endplate. Further studies will be aimed at studying the structure/function of Sorbs1 in synaptic differentiation. The current study extends the early signaling requirement downstream of agrin stimulation from MuSK/Dok7/Crk-CrkL to now include Sorbs1/2.

## Supplementary Material

Supplemental material
