# Supplementary material for: Sorbs1 and -2 Interact with CrkL and Are Required for Acetylcholine Receptor Cluster Formation
Source: Mol Cell Biol. 2016 Jan 4;36(2):262–70. doi: 10.1128/MCB.00775-15 (PMC4719301; doi:10.1128/MCB.00775-15)
Supplement: Supplemental material [file supp_36_2_262__index.html]

Sorbs1 and -2 Interact with CrkL and Are Required for Acetylcholine Receptor Cluster Formation — Supplemental material 

# Sorbs1 and -2 Interact with CrkL and Are Required for Acetylcholine Receptor Cluster Formation

## Supplemental material

- Supplemental file 1 -

  Legends to Table S1 and Data Set S1

  PDF, 50K
- Supplemental file 2 -

  Table S1 (Most abundant CrkL binding proteins in myotubes)

  PDF, 862K
- Supplemental file 3 -

  Data Set S1 (MS/MS data set for CrkL binding proteins)

  XLSX, 371K
