# Supplementary material for: Sorbs1 and -2 Interact with CrkL and Are Required for Acetylcholine Receptor Cluster Formation
Source: Mol Cell Biol. 2016 Jan 4;36(2):262–70. doi: 10.1128/MCB.00775-15 (PMC4719301; doi:10.1128/MCB.00775-15)
Supplement: Supplemental material [file MCB.00775-15_zmb999101084so1.pdf]

Supplemental information

Sorbs1 and 2 interact with Crk-L and are required for Acetylcholine receptor cluster formation

Peter T. Hallock, Sherry Chin, Steven Blais, Thomas A Neubert, and David J. Glass

Supplemental Legends

Table 1

Most abundant CrkL binding proteins in myotubes.

A summary of the most abundant CrkL binding proteins based on absolute intensity based quantitation (IBAQ). The table shows gene name and relative abundance. Additionally, total peptides identified in each condition and how many of those peptides are unique are reported. Finally, the percentage of protein coverage represented by each of the unique peptides is reported in the final column.

Supplemental File 1

Complete MS/MS dataset for CrkL binding proteins
