# Supplementary material for: Sorbs1 and -2 Interact with CrkL and Are Required for Acetylcholine Receptor Cluster Formation
Source: Mol Cell Biol. 2016 Jan 4;36(2):262–70. doi: 10.1128/MCB.00775-15 (PMC4719301; doi:10.1128/MCB.00775-15)
Supplement: Supplemental material [file MCB.00775-15_zmb999101084so2.pdf]

Table 1

Abundance	Protein Identification	Total Peptides Sham:CrkL	Unique Peptides	Coverage (%)
1	CrkL	3 : 24	3 : 24	87.1
2	Actin	17 : 27	4 : 4	79
3	C3G	0 : 37	0 : 1	35.1
4	Tubulin	11 : 18	1 : 2	43.8
5	Arhgef5	0 : 63	0 : 63	40
6	Asap1	0 : 34	0 : 33	36.1
7	Sos1	0 : 45	0 : 39	36.8
8	Tns1	1 : 34	0 : 33	27.9
9	Abl1	0 : 28	0 : 23	28.2
10	Grb2	1 : 8	1 : 8	30.4
11	Asap3	0 : 15	0 : 15	21.8
12	Pik3cb	0 : 19	0 : 19	17.6
13	Abl2	0 : 22	0 : 1	27
14	Eps15	0 : 24	0 : 24	40.5
15	Ship2	5 : 22	5 : 22	23
16	Coro6	1 : 6	1 : 6	12.7
17	Pragmin	0 : 19	0 : 19	16.5
18	LL5beta	0 : 26	0 : 26	23.2
19	Dok-7	0 : 8	0 : 8	18.1
20	LL5alpha	0 : 29	0 : 3	23.8
21	SgK269	0 : 38	0 : 38	27.8
22	Sos2	0 : 29	0 : 23	21.8
23	Nckipsd	0 : 13	0 : 13	18.3
24	Dock5	0 : 51	0 : 50	37.3
25	Elmo2	0 : 15	0 : 11	21.8
26	Usp7	0 : 15	0 : 1	13.7
27	Pik3r1	0 : 20	0 : 18	28.2
28	Cttnbp2nl	0 : 17	0 : 17	29.5
29	Dock1	0 : 37	0 : 36	26.1
30	Map4k5	0 : 16	0 : 15	20.4
31	Ppp1ca	0 : 6	0 : 4	15.8
32	Phactr4	0 : 13	0 : 13	26.7
33	Arhgap42	0 : 19	0 : 19	26.9
34	Uso1	1 : 11	1 : 11	15
35	Cytsa	2 : 16	2 : 16	15.4
36	Anln	0 : 14	0 : 14	17.8
37	Ogdh	1 : 11	1 : 11	13.6
38	Pik3r2	0 : 17	0 : 15	23.8
39	Tks5	1 : 8	1 : 8	10.1
40	Ddb1	0 : 8	0 : 8	9.4
41	Cltb	0 : 5	0 : 5	17.9
42	Mapk1	0 : 5	0 : 5	11.7
43	Sorbs2	0 : 2	0 : 2	43.9
44	Tks4	0 : 5	0 : 5	6.7
45	Psmd1	1 : 10	1 : 10	15.4
46	Hnrnpf	0 : 5	0 : 3	18.3
47	Megf10	0 : 4	0 : 4	4.8
48	Gtf2i	0 : 8	0 : 8	11.7
49	Tenc1	1 : 10	0 : 9	10.2
50	Rai14	0 : 11	0 : 11	13.9
51	Sash1	0 : 9	0 : 9	9.3
52	Mtap4	2 : 10	2 : 10	13.9
53	Arhgef2	0 : 10	0 : 10	12.6
54	Rin3	0 : 9	0 : 9	11.7
55	Ppfibp2	0 : 14	0 : 12	17.5
56	Dlgap4	0 : 9	0 : 8	12.4
57	Ubr3	0 : 7	0 : 7	4.8
58	Synpo2L	0 : 9	0 : 9	13
59	Sntb1	0 : 8	0 : 7	17.1
60	Utrophin	0 : 38	0 : 38	12.8
61	wdr70	0 : 6	0 : 6	11.3
